# Supplementary material for: Biorefinery of Tomato Leaves by Integrated Extraction and Membrane Processes to Obtain Fractions That Enhance Induced Resistance against Pseudomonas syringae Infection
Source: Membranes (Basel). 2022 May 31;12(6):585. doi: 10.3390/membranes12060585 (PMC9229720; doi:10.3390/membranes12060585)
Supplement: Supplementary file 1 [file membranes-12-00585-s001.zip › membranes-1735547-supplementary.pdf]

Article

Supplementary Materials:

Biorefinery of Tomato Leaves by Integrated Extraction and Membrane Processes to Obtain Fractions That Enhance Induced Resistance against *Pseudomonas syringae* Infection

Fabio Bazzarelli [1], Rosalinda Mazzei [1,*], Emmanouil Papaioannou [2,*], Vasileios Giannakopoulos [3], Michael R. Roberts [3] and Lidietta Giorno [1]

1 National Research Council of Italy, Institute on Membrane Technology, CNR-ITM, via P. Bucci 17 C, 87036 Rende, Italy; f.bazzarelli@itm.cnr.it (F.B.); r.mazzei@itm.cnr.it (R.M.); l.giorno@itm.cnr.it (L.G.)
2 Engineering Department, Lancaster University, Lancaster, LA1 4YW, UK; e.papaioannou@lancaster.ac.uk (E.P.)
3 Lancaster Environment Centre, Lancaster University, Lancaster, LA1 4YQ, UK; v.giannakopoulos@lancaster.ac.uk (V.G.); m.r.roberts@lancaster.ac.uk (M.R.R.)
* Correspondence: r.mazzei@itm.cnr.it (R.M.); e.papaioannou@lancaster.ac.uk (E.P.)

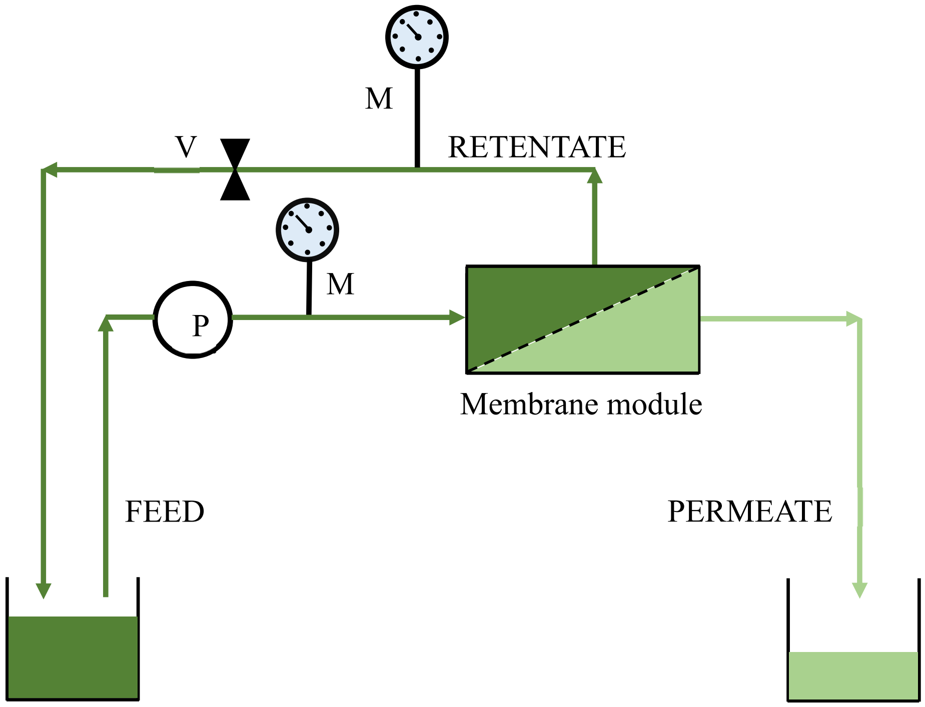

Figure S1. Membrane process (M: manometer, P: pump, V: valve)
